# Affording introspection: an alternative model of inner awareness

**DOI:** 10.1007/s11098-014-0421-x

**Published:** 2014-12-20

**Authors:** Tom McClelland

**Affiliations:** School of Social Sciences, University of Manchester, Oxford Road, Manchester, M13 9PL England

**Keywords:** Consciousness, Inner awareness, Introspection, Subjectivity, Phenomenology, Affordances

## Abstract

The ubiquity of inner awareness thesis (UIA) states that all conscious states of normal adult humans are characterised by an inner awareness of that very state. UIA-Backers support this thesis while UIA-Skeptics reject it. At the heart of their dispute is a recalcitrant phenomenological disagreement. UIA-Backers claim that phenomenological investigation reveals ‘peripheral inner awareness’ (or ‘pre-reflective self-consciousness’) to be a constant presence in their non-introspective experiences. UIA-Skeptics deny that their non-introspective experiences are characterised by inner awareness, and maintain that inner awareness is only gained when they explicitly introspect. Each camp has put forward a range of arguments designed to resolve this dispute, but I argue that none of these arguments has genuine dialectical purchase. This leads me to develop a compromise position that trades on the contribution that affordances can make to our phenomenology. According to the Affordance Model of inner awareness, all conscious states of normal adult humans are characterised by an affordance of introspectability. In line with the UIA-Skeptic, non-introspective experiences are not characterised by inner awareness. But against the traditional UIASkeptic, non-introspective experiences are characterised by an awareness of the opportunity for introspection. On this view, our capacity to gain inner awareness of our current experience is a ubiquitous feature of our phenomenology. I show how the Affordance Model respects the driving phenomenological intuitions of both the UIA-Backers and the traditional UIA-Skeptics, and suggest that it is able to explain why neither camp achieves an accurate description of how inner awareness figures in their phenomenology.

## A recalcitrant phenomenological disagreement

### Is inner awareness ubiquitous?

Is your conscious ‘outer awareness’ of the world always accompanied by a conscious ‘inner awareness’ of that very experience? This is a question about your phenomenology—about what it’s like to undergo the experience you are having. The same question can be expressed in a number of different ways: when you experience some event do you also experience your experience?; do your conscious states involve a *self*-consciousness of that very state?; when you are perceptually aware of your environment, are you also aware of your awareness? Introspection certainly seems to yield awareness of one’s own experience, but the question is whether your *non*-introspective experiences are characterised by a kind of inner awareness too. One camp answers ‘yes’ to these questions: they report that inner awareness is a ubiquitous feature of their phenomenology. The other camp answers ‘no’ to these questions: they report that their phenomenological reflection reveals no such ubiquitous property.

One possibility here is that both sides are getting their own phenomenology right: the yes-camp has one kind of experience, the no-camp has a different kind, and both camps are accurately reporting what their own experiences are like. Although this suggestion has the attraction of making everybody right, I think all sides can agree that it is wildly implausible. If there is such a thing as this non-introspective inner awareness, why would it be a ubiquitous feature of some people’s experience but not others? The more likely situation is that both camps have the same kind of experience but at least one camp is describing their phenomenology inaccurately. So the real question is whether the following phenomenological thesis is true:

**The Ubiquity of Inner Awareness Thesis (UIA):** all conscious states of normal adult humans are characterised by an inner awareness of that very state.

Those who affirm this phenomenological thesis are UIA-Backers. Those who do not affirm this thesis, including those who actively deny it, are UIA-Skeptics.^1^ The UIA-Backers and UIA-Skeptics offer conflicting views of what our conscious experience is like.

UIA holds that we are always aware of our concurrent experience. It is important to recognise that this thesis does not place demanding constraints on the *way* in which we are aware of our experience. Our inner awareness does not, for instance, need to be an *attentive* awareness. Nor need it involve the application of *concepts* to our own experience.^2^ Nor does it have to be an *objectual* awareness—that is, an awareness that presents its target as an entity that exists independently of our being aware of it. It may not even require an *egological* representation of ourselves as the subjects of that experience—a state might be self-conscious in the sense of yielding consciousness *of itself* without being self-conscious in the sense of yielding consciousness of *the subject that bears it*.^3^ So if our inner awareness is *inattentive, non*-*conceptual, non*-*objectual* and *non*-*egological* that would be quite consistent with the truth of UIA. One constraint that UIA *does* place on our inner awareness is that it be *conscious* awareness. Some take ‘awareness’ to be synonymous with ‘consciousness’ but others allow awareness to come in non-conscious forms. The ubiquity of non-conscious inner awareness—a possibility I will consider in due course—would not entail the truth of UIA. By ‘awareness’ I shall mean conscious awareness. Some of the more demanding features mentioned might be required for *introspective* awareness of one’s experience, but UIA does not claim that all experiences are introspected.^4^ Kriegel holds that introspection brings into focal attention an inner awareness that is otherwise only a peripheral aspect of our experience (Kriegel 2009a, p. 183). Zahavi draws attention to a crucial distinction made in the phenomenological tradition between reflective and pre-reflective self-consciousness (e.g. 2006, p. 5). *Reflective* self-consciousness is achieved only when we introspect, but the suggestion is that there is a more primitive *pre*-reflective self-consciousness that is a constant background presence in experience.^5^

### Theoretical implications of UIA

UIA is an interesting thesis about the phenomenology of normal adult humans. More than that though, it has significant implications for our theorising about consciousness. Importantly, some have used UIA as the basis for bolder claims about the nature of consciousness. First, one might draw an abductive inference from UIA to the *unrestricted* thesis that all possible conscious experiences are characterised by an inner awareness of that very state. On this view, UIA offers us an insight into the invariant structure of conscious experience, allowing us to conclude that the experiences of abnormal adult humans, of human infants and of conscious non-human animals must also all be characterised by inner awareness. Second, from this unrestricted phenomenological thesis one might draw an inference to the *metaphysical* conclusion that inner awareness is what makes conscious mental states conscious at all. This comes out vividly in Kriegel’s self-representationalist theory of consciousness according to which a mental state is conscious in virtue of suitably representing itself (Kriegel 2009a). Neither of these inferences is beyond reproach. Mehta, for instance, casts serious doubt on both (2013, pp. 361–363). But the fact that UIA is used to drive such substantive theoretical claims gives us all the more reason to assess its credibility.

Even if UIA is not used to *motivate* theoretical claims about consciousness, it can certainly be used to *test* such claims. Higher-order representation (HOR) theories claim that we are aware of a mental state M only when we suitably represent it via some distinct higher-order mental state M* (e.g. Rosenthal 1986). Some advocates of UIA have suggested that HOR theories are incompatible with UIA (Kriegel 2009a, pp. 124–125). If UIA is true, we are not just aware of M but also of our *awareness of* M. To accommodate this the HOR theorist might posit some third mental state M** in virtue of which we are aware of M* representing M. But this merely pushes the problem up a level, leaving us unaware of the awareness bestowed by M**. Assuming we can rule out an infinite hierarchy of mental states, it looks like the truth of UIA is at odds with the truth of HOR theory.

‘One-state’ theorists have attempted to avoid this objection whilst maintaining something of the spirit of HOR theory. Echoing a move made by Brentano (1874/1924), they suggest that consciousness involves a single mental state that represents both the world *and itself*. The hope is that such reflexive representations can yield awareness of our own awareness. This strategy has been developed in a number of competing ways, and the resulting theories can again be tested by asking whether they accommodate the truth of UIA. Kriegel, for instance, suggests that this reflexive representation is achieved when a lower- and higher-order mental state appropriately combine to form a single complex mental state. But there is a genuine worry that this kind of internal representational division would entail the same kind of regress that threatens HOR theory.^6^

The truth of UIA would be a crucial datum for theorising about consciousness. Conversely, if UIA is false this too would have important theoretical consequences, cutting off any lines of argument premised on UIA. My aim in this paper is not to evaluate theoretical claims about consciousness, nor to assess the validity of any inferences made from UIA to some theoretical conclusion. Rather, my aim is to look closely at this putative phenomenological datum itself, putting aside any theoretical matters that might cloud our assessment.

### Strategies for resolving the dispute

UIA-Backers claim to have phenomenological justification for their belief in UIA.^7^ Kriegel holds that ‘…peripheral inner awareness is simply phenomenologically manifest’ (2009a, p. 50) and Zahavi suggests that accurate phenomenological description is ‘the best argument to be found’ for thinking that non-introspective experience is characterised by pre-reflective self-consciousness (2006, p. 24). But UIA-Skeptics claim not to find any such feature in their experience. Mehta reports ‘[w]hen I conduct phenomenological investigation into any peripheral awareness of my experiences, attempting to set aside any theoretical prejudices, I haven’t a clue whether I’ve ever had such peripheral awareness’ (2013, p. 364). And Gennaro states ‘[i]t does not seem to me that I am consciously aware (in any sense) of my own experience when I am, say, consciously attending to a play or the task of building a bookcase’ (2008, p. 48). Schear (2009, p. 100) reports that his polling on the matter reveals no consensus for or against UIA and my own informal polling reveals much the same.

Faced with this recalcitrant phenomenological disagreement, what can we do to resolve the dispute? Two broad strategies present themselves. The *via* *positiva* strategy is to establish *indirect* evidence for UIA. On this strategy, UIA-Backers identify particular features of experience—features that their opponents can agree are genuine—and then argue that UIA is the best explanation of these features. The challenge for UIA-Skeptics is to resist these inferences. The *via negativa* strategy is to explain why one side has got their own phenomenology wrong. The task for the UIA-Backers is to explain why the UIA-Skeptics have failed to recognise a ubiquitous feature of their phenomenology, while the task for the UIA-Skeptics is to explain why the UIA-Backers are under the illusion that their experiences have a property that they in fact lack.

In Sect. 2 I explore the three leading implementations of the *via positiva* strategy. I argue that, in all three cases, the UIA-Skeptics can offer their own credible explanations of the data cited without having to posit UIA. Moving on to the *via negativa* strategy, I consider four proposals put forward by UIA-Backers designed to explain away skepticism about UIA, and four proposals put forward by UIA-Skeptics designed to explain away any attraction to UIA. I argue that no proposal takes seriously enough the intuitions of the opposing camp. I conclude that none of the moves made in the dispute over UIA have the dialectical purchase needed to break the phenomenological stalemate.

In Sect. 4 I introduce my own model of inner awareness which offers a novel alternative to the entrenched positions in this debate. I suggest that all conscious experiences of normal adult humans are characterised by an *affordance of introspectability*. That is, ordinary non-introspective experience does *not* involve the subject being aware of her experience but *does* involve the subject being aware of her potential to become aware of her experience. This ‘Affordance Model’ denies UIA, officially putting me in the UIA-Skeptic camp. Unlike the established skeptical positions though, the Affordance Model reflects something of the spirit of UIA. As such I regard it as a compromise position that, instead of simply adopting a hard-line stance, takes seriously the intuitions of both camps. Furthermore, I argue that this model is much better equipped than hard-line views to explain why its opponents have got their own phenomenology wrong.

## The *via positiva* strategy

### Three *via positiva* arguments for UIA

(i) *Reportability* Whenever we are in a conscious mental state, we are easily able to report on that very mental state when prompted.^8^ This is a datum that the UIA-Skeptic ought to accept: it is a highly plausible claim about consciousness, and its plausibility does not rely on any presupposition that UIA is true. UIA-Backers argue that the best explanation of this datum is that all consciousness includes a peripheral/pre-reflective consciousness of itself. Zahavi, drawing explicitly on Sartre, argues as follows:

If I am engaged in some conscious activity, such as the reading of a story, my attention is neither on myself nor on my activity of reading, but on the story. But if my reading is interrupted by someone asking me what I am doing, I reply immediately that I am (and have for some time been) reading; and the self-consciousness on the basis of which I answer the question is not something acquired at just that moment but a consciousness of myself which has been present to me all along. (2004, p. 84)

Since the experience is one of immersion in the story, it would be implausible to explain the report by saying that the subject was explicitly introspecting his experience whilst reading. Since the report is immediate and effortless, it would be implausible to say that the report was made on the basis of an introspection performed by the subject after he was interrupted. We are thus encouraged toward the conclusion that the subject’s experience of the story is characterised by a non-introspective awareness of that very experience. And since we (normal adult humans) are *always* able to report on our conscious states, we can infer that our experiences are *always* characterised by this inner awareness.^9^ So regardless of her prior phenomenological intuitions, the UIA-Skeptic ought to infer that UIA is true after all.

(ii) *No Surprises* At present you are, I assume, visually perceiving the words before you. Now start to introspect. What do you find? Presumably you find that you are undergoing a visual experience as of reading the words before you. I imagine that this doesn’t come as news to you—*I’m* not surprised that you find this experience on introspecting so it would be very odd if *you* were surprised! This is representative of a general truth about introspection: when we introspect we are unsurprised to find just the experience we do. Perhaps introspection can reveal specific properties of our experience that are surprising, but it remains the case that finding the experience itself never feels like a substantive discovery.

This is a datum about consciousness—specifically about introspective consciousness—that the UIA-Skeptic ought to accept. UIA-Backers suggest that the best explanation of this datum is that we are already aware of our experience prior to introspecting it. Kriegel explains that ‘[i]f conscious states were states we were aware of, then they would not be surprising to notice when we turned our attention onto them’ (2009a, p. 307). Introspection cannot surprise us because it doesn’t make us aware of anything we weren’t aware of prior to introspecting. On Kriegel’s view, introspection simply brings into focus something of which we were already aware peripherally. Or, in Zahavi’s framework, introspection brings into reflective awareness that which was already given to us pre-reflectively. Thus the ‘no surprises’ datum ought to convince the UIA-Skeptic that UIA is true.

(iii) *Nothing Dramatic* The last *via positiva* argument I will consider is based on an observation about how it feels to introspect. When you make the transition from perceiving your current environment to introspecting your current perceptual experience, does it feel as though you are introducing a wholly new representation into your experience? Most would say that introspection feels less dramatic than this. Kriegel holds that ‘…introspecting feels more like a phenomenologically light shifting around of attention than like a dramatic mental act that produces a completely new awareness’ (2009a, p. 186). Introspection is not like opening our eyes, revealing visible objects of which we were previously unaware. Rather, introspection is more like visually attending to an object that we were previously seeing inattentively.

The UIA-Backers explain this datum by saying that introspection *really doesn’t* produce any new awareness: it *seems* undramatic because it *is* undramatic. Introspecting is a modest shifting around of attention, or a subtle transition from pre-reflective to reflective awareness. In other words, introspection merely enhances the inner awareness that is already contained in our non-introspective experience. So given this datum about how the act of introspection feels, the UIA-Skeptic ought to infer that UIA is true.^10^

### The skeptical response

How can the UIA-Skeptic respond to these *via positiva* arguments? One option is to reject the putative data, but this would come at the cost of having little dialectical force against those who find the data plausible. A better route for the skeptic is to find a way of accommodating the data whilst avoiding UIA. UIA may well explain the data cited, but is it the *best* explanation? If the UIA-Skeptic can provide explanations of the data that are at least as plausible as those provided by the UIA-Backer, then the inference to UIA can be blocked.^11^

(i) *Reportability* The fact that we can report on our concurrent conscious activity indicates that we have *knowledge* of our conscious states as we undergo them. Claiming, as the UIA-Backer does, that we are *aware* of our concurrent conscious state is one way of accounting for this epistemic situation. But it is not the only way. At any given time, we have a great deal of non-conscious knowledge—things that we know but which do not figure in our experience. As Schear explains:

…the move from an epistemological self-relation (knowing what I am doing in doing what I am doing) to a phenomenological self-relation (experiencing myself doing what I am doing in doing what I am doing) is a move, and it is not an obvious move to make. (2009, p. 101)

Avoiding this unwarranted move, the UIA-Skeptic can explain the reportability of conscious states by saying that our conscious states are accompanied by a concurrent *non*-conscious knowledge of that very state (Thomasson 2005). Here we have a perfectly credible explanation of the data cited that involves no appeal to inner awareness.^12^

(ii) *No Surprises* Once the UIA-Skeptic has posited an ever-present non-conscious knowledge of our concurrent conscious state, she is also in a position to accommodate the ‘no surprises’ datum. When we introspect we are unsurprised by the experience we find because we already knew about that experience prior to introspection. The UIA-Backer assumes that this knowledge takes the form of inner awareness, but the UIA-Skeptic can reject that assumption and hold that the relevant knowledge is non-conscious.^13^ Consider again the introspection of your perceptual experience of the words before you. This perceptual experience is unsurprising to you because you already knew you were having this experience before you began to introspect. But since this knowledge was non-conscious, your non-introspective experience of the words was exhausted by your outer awareness of the world. There is no need to infer that inner awareness was contributing to your phenomenology.^14^

(iii) *Nothing Dramatic* A UIA-Skeptic might adopt a model of introspection according to which we observe our own experiences when, and only when, we begin to introspect (e.g. Armstrong 1968). The fact that introspection does not feel like such a ‘dramatic’ act would count against such a view. Most UIA-Skeptics, however, would not wish to adopt such a model of introspection. A number of more promising models of introspection are available according to which introspection is, in the relevant sense of the term, an *un*dramatic act. For instance, Thomasson draws on the influential Husserlian notion of bracketing:

…the idea of bracketing in phenomenology is to preserve both force and content of the original experience…but use the brackets to disconnect it from our ordinary world-directed concern so that it can be studied as a phenomenon, a way of experiencing the world, rather than being put to use in our engagement with the world… (2005, p. 125)

She goes on to describe this transformation of our consciousness as ‘…a *shift of attitude* within our experiences: regarding them merely as *appearances*, as *representing contents*, rather than simply *using* them to acquire information about the world’ (2005, pp. 126–127).^15^ If accurate, this would explain why introspection does not feel dramatic without presupposing that inner awareness is present in the background of our non-introspective experiences.^16^ The UIA-Skeptic need not commit herself to the Husserlian model of phenomenological knowledge here. Rather, she can make the weaker claim that there is no need to posit UIA to explain the ‘nothing dramatic’ datum since alternative models are available with just as much explanatory promise.^17^

The *via positiva* strategy has failed to tip the balance in favour of UIA. Although credible explanations of the data cited can be provided by UIA, it is far from clear that these are the *best* explanations as plausible competing explanations are available that do not posit UIA. As such, the UIA-Backer has said nothing that ought to convince the UIA-Skeptic to switch sides. In fact, it seems that preferences for one explanation over another are governed by one’s *prior* attitude toward the truth of UIA. So, far from providing an objective way of resolving the dispute, the *via positiva* strategy has no real dialectical purchase, leaving us with the same recalcitrant phenomenological disagreement with which we started.^18^ Although my review of the *via positiva* strategy is far from comprehensive I think it offers a fair assessment of what the literature has to offer, so for the remainder of the paper I will assume that no existing version of the strategy succeeds.

## The *via negativa* strategy

The aim of the *via negativa* strategy is to establish a credible explanation of why the opposing camp has ended up misdescribing their own phenomenology. A successful implementation of this strategy must cast serious doubt on the opposing position and ought to shake opponents’ confidence in their phenomenological reports. This strategy does not seem to have been implemented with any great enthusiasm by UIA-Backers. Kriegel introduces four quick considerations designed to explain away resistance to UIA (2009a, pp. 51–52), but I will argue that none of these considerations has any real force against the UIA-Skeptic. The strategy has been adopted much more enthusiastically by UIA-Skeptics. I will focus on arguments put forward by Thomasson (2006), Gennaro (2008), Schear (2009) and Mehta (2013), and again will conclude that they fail to break the dialectical stalemate between the two camps.

One option available to both sides is to say that their opponents are simply reflecting on their phenomenology badly. But this argument would clearly lack any dialectical purchase as the opposing camp can simply turn the same argument around. Furthermore, since it is implausible that an opponent is a bad phenomenological investigator *across the board*, it is unwarranted to suggest that she is making a careless mistake in the case of her investigation of inner awareness. I think two general lessons can be extracted from this:

(I) A successful *via negativa* argument cannot be applicable against both camps with equal force.
(II) A successful *via negativa* argument cannot attribute to the opponent a kind of mistake that she is not disposed to make in contexts other than the assessment of UIA.

Although these two rules are quite demanding, a proper appreciation of what is required of the *via negativa* strategy reveals them to be appropriate. Consider a scenario in which we have a compelling positive theoretical argument for our preferred position. This would leave us with the residual task of explaining why our opponent has got things wrong. In such a situation, the demands placed on our explanation would be relatively weak. It wouldn’t matter, for instance, if our opponent could generate an inverted explanation of why *our* intuitions are wrong because we would not be relying on our intuitions to make our case: rather, our intuitions would be backed up by a compelling theoretical argument. It is important to recognise that this is *not* the scenario in which we currently find ourselves. I have argued already that theoretical considerations do not resolve the debate. Consequently, the *via negativa* strategy needs to provide a *self*-*standing argument* for one’s preferred position. In light of this, the two demanding rules I have introduced are quite appropriate. I will argue that *none* of the *via negativa* arguments that have been put forward manage to satisfy both rules.

### Four *via negativa* arguments against the UIA-Skeptic

(i) *Unimpressiveness* Kriegel notes that ‘…peripheral inner awareness is not as phenomenologically impressive as, say, the qualitative character of color experience. But the common tendency to take color experiences as the gold standard of phenomenology…may set the bar too high’ (2009a, p. 51). The suggestion is that UIA-Skeptics fail to notice the inner awareness that characterises their experience because it is such a subtle feature. This may well explain why inner awareness is *harder* to spot than more striking aspects of our phenomenology, such as the reddish quality of our visual experience. But why should we think that the UIA-Skeptic has carelessly failed to consider the more subtle aspects of his phenomenology when he searches for inner awareness? After all, the UIA-Skeptic isn’t inclined to deny the existence of *other* subtle aspects of his phenomenology. Gennaro, for example, reports that a peripheral awareness of his thoughts makes a subtle contribution to his phenomenology (2008, p. 44), so why would he fail to recognise the subtle presence of peripheral inner awareness? The argument from unimpressiveness breaks the second rule identified above: it attributes to the UIA-Skeptic a kind of mistake—in this case a failure to recognise unimpressive features of their phenomenology—that he is not disposed to make in contexts other than the examination of inner awareness. Perhaps Kriegel could *supplement* his argument with an explanation of why the UIA-Skeptic makes this mistake specifically in the context of inner awareness, but it would then be this supplementary move that is doing the real philosophical work, rendering the appeal to unimpressiveness somewhat redundant.

(ii) *Unintrospectability* Kriegel’s next line of argument is that we normally identify features of our phenomenology by attending to them. In the case of peripheral inner awareness though, ‘…if we attempt to turn our attention to it, it inevitably transforms into focal inner awareness’ (2009a, p. 52). We can translate the same point into Zahavi’s framework: our pre-reflective self-consciousness cannot be revealed by explicit reflection on our experience, because such reflection *transforms* our self-consciousness from pre-reflective to reflective. If inner awareness is unintrospectable, how do UIA-Backers like Kriegel know that it is there? Kriegel suggests that we have ‘…a general impression of peripheral inner awareness from our ordinary, non-introspective consciousness’ (2009b, p. 375). This account might explain why peripheral inner awareness is harder to spot than features of phenomenology that can simply be attended to explicitly. Again though, the problem is that UIA-Skeptics don’t generally make the mistake of denying the existence of inattentive aspects of their phenomenology. Mehta, for instance, acknowledges that he has an inattentive awareness of his keyboard as he types (2013, p. 364). As such, Kriegel still owes us an explanation of why the UIA-Skeptic would make such a mistake when it comes to peripheral inner awareness.

(iii) *Background Hum* Kriegel notes that ‘[i]t is in general difficult to notice even stimuli that are constant for a relatively short time, such as the hum of the refrigerator pump. If peripheral inner awareness is indeed ubiquitous, its constancy throughout our waking life would account for the fact that it is so phenomenologically elusive’ (2009a, p. 52). Our inner awareness is thus like a humming fridge that *never* stops. Since we never have an experience *lacking* this phenomenological feature, we are unable to contrast our conscious states and bring our peripheral inner awareness ‘into sharper relief’ (2009a, p. 52). The problem with this line of argument should be clear: although it might explain why peripheral inner awareness is harder to spot than features that vary between experiences, this does not explain the UIA-Skeptic’s mistake. Presumably the skeptics aren’t such careless phenomenologists that they would deny the existence of the background hum of the fridge, even when prompted by opponents who claim that such a humming is present. So why would they be making an analogous mistake in the case of inner awareness?

Overall, Kriegel’s attempts to implement the *via negativa* strategy are inadequate. They may well explain someone’s initial resistance to UIA—which is, to be fair, all Kriegel claims they succeed in doing (2009a, p. 52)—but they cannot explain the mistake of a *considered* UIA-Skeptic who has carefully examined her experience but is still unable to find the putative background hum of inner awareness. Kriegel’s arguments fail to satisfy the second rule: they attribute to the skeptic a kind of mistake when reflecting on inner awareness that she is not otherwise disposed to make.

### Four *via negativa* arguments against the UIA-Backer

Many UIA-Skeptics are acutely aware of the need to account for what they take to be the phenomenological mistake of their opponents (Thomasson 2006, p. 7; Gennaro 2008, pp. 48–49; Schear 2009, p. 101). Mehta, for instance, asks ‘…how could Kriegel and those like him have gotten their phenomenology wrong?’ (2013, p. 365). I consider the four main answers offered to this question but find them all wanting. The first and second fail to offer arguments that cannot be turned around with equal force by the UIA-Backer. The third and fourth attribute to the UIA-Backer a kind of mistake that he is not otherwise disposed to make.

(i) *Theoretical Bias* One of the main problems we face when assessing the veracity of a subject’s phenomenological reports is that those reports can be influenced by the background theoretical commitments of the subject. As outlined in Sect. 1, UIA is bound up with a number of theoretical claims about consciousness. The suggestion is that UIA-Backers have particular theoretical beliefs that skew the way they regard their own experience.^19^ Kriegel, for instance, claims that his unbiased phenomenological commitment to UIA drives his Self-Representationalist theory of consciousness. But perhaps, instead, it is a background theoretical commitment to Self-Representationalism that underwrites the convictions he has about his phenomenology. On this account, the theoretical judgment that one’s experience *must* always be characterised by inner awareness leads to the erroneous phenomenological judgement that one’s experience *is* always characterised by inner awareness.

It is hard to deny that theoretical commitments sometimes lead one’s phenomenological judgements astray, so to that extent the argument from theoretical bias has some credibility. Despite this, it is not an argument that will help us break the phenomenological stalemate at hand. The problem is that all parties are coming into the discussion of UIA with certain background theoretical opinions about consciousness. So although it is credible that UIA-Backers are making theoretically biased phenomenological judgements, it is *equally* credible that it is the judgements of the UIA-Skeptic that are biased. Some claim, for example, that inner awareness requires a reflective act (e.g. Thomasson 2005). Perhaps, then, the conviction that non-introspective experiences *ought* not to be characterised by inner awareness leads UIA-Skeptics to the erroneous phenomenological judgement that their experiences *are* not so characterised. In other words, the argument from theoretical bias cuts both ways, so cannot tip the balance of the debate one way or the other.

(ii) *The Fridge Light Fallacy* Whenever you open the fridge door you find that the fridge light is on. From this you might infer that the fridge light is *always* on, even when the door is closed. But, of course, this inference would be mistaken as it is the act of opening the door that makes the light turn on. The proposal is that investigating whether we are aware of our conscious states can lead us to make an analogously fallacious inference. Schear argues:

…to reflect on the structure and character of our own experience is an intensely self-conscious enterprise. As soon as we’ve set off on the investigation, we’ve ‘‘opened the refrigerator.’’ Unsurprisingly, self-consciousness turns up wherever we look. And then we proceed to call it ‘‘pre-reflective’’ to ease the pangs of our guilty phenomenological conscience. (2009, p. 101)

Whenever we introspect we find an experience that is clearly characterised by an inner awareness of that very experience. From this, the UIA-Backer infers that his non-introspective experiences must also involve inner awareness, albeit in an attenuated form. But what he should have recognised is that the very act of introspection is what *introduced* inner awareness into his experience, meaning the inference is mistaken.^20^

As with the argument from theoretical bias, this proposal identifies a kind of mistake to which we are genuinely vulnerable. Again though, the argument fails to break the phenomenological stalemate. UIA-Backers can simply reverse the argument and suggest that UIA-Skeptics fail to recognise that introspection ‘switches off’ our peripheral/pre-reflective awareness. If UIA is true, there is a pre-reflective inner awareness that all our non-introspective experiences have. Perhaps the UIA-Skeptic makes the following mistake: whenever she introspects, she finds only focal/reflective inner awareness and never the peripheral/pre-reflective inner awareness posited by the UIA-Backer. From this she infers that *none* of her experiences are characterised by pre-reflective inner awareness. But what she should have recognised is that the act of introspection is what removed the peripheral/pre-reflective inner awareness from her experience, replacing it with focal/reflective inner awareness. This is effectively the thrust of Kriegel’s *via negativa* argument from unintrospectability discussed above. Yet again, the phenomenological stalemate persists.

(iii) *The Non*-*Conscious/Conscious Confusion* The UIA-Skeptic is free to grant that whenever we are in a conscious state, we have a concurrent knowledge of that conscious state. So long as the knowledge is itself *non*-*conscious*, this entails no commitment to UIA. Perhaps the UIA-Backer is then making the following error: he correctly recognises that he always has knowledge of his concurrent conscious state, but mistakes this *non*-conscious knowledge for *conscious* knowledge. In other words, when the UIA-Backer reports that inner awareness is a ubiquitous feature of his experience, he is reading his non-conscious knowledge into his phenomenology. This suggestion comes out explicitly in the passages from Schear discussed in Sect. 2.2.^21^ One of the attractions of this proposal is that it does not uncharitably suggest that the UIA-Backer is entirely misguided in his support of UIA. Rather, he has latched onto a genuine truth about the epistemic status of consciousness, but then made a mistake about how best to understand this epistemic status.

The problem with this proposal is that UIA-Backers are not generally inclined to confuse conscious and non-conscious knowledge. At any given time, Uriah Kriegel knows his name and could report what his name is when asked. But he is not (I assume) inclined to read this non-conscious knowledge into his experience: he does not posit a ubiquitous peripheral name-awareness to explain his epistemic situation. So why would he be making an analogous mistake in the case of his non-conscious knowledge of his concurrent conscious state? As it stands, this argument fails to offer a credible explanation of the UIA-Backer’s mistake. At best, it is an incomplete proposal that needs to be supplemented with an account of why the UIA-Backer is vulnerable to this mistake in the case of knowledge of consciousness and not other cases of knowledge.

(iv) *The Disposition/Actualisation Confusion* The UIA-Skeptic is free to grant that all conscious states are introspectable, or more cautiously that all typical conscious states of normal adult humans are introspectable. On this view, whenever a subject is conscious she has the *potential* to gain an inner awareness of their concurrent conscious state. But this disposition is only *actualised* when we explicitly introspect, never during our ordinary non-introspective experience.^22^ The suggestion is that UIA-Backers confuse this constant dispositional property of experience with the actualisation of that disposition, leading them to report that inner awareness is a constant *actual* presence in their experience. Thomasson argues that ‘…it is our ability to have immediate first-person knowledge of our own experiences … that leads many to think of them as states we are continually aware of’ (2006, p. 8). Similarly, Schear hypothesises that UIA-Backers are guilty of the ‘category mistake’ of ‘…construing the presence of a capacity for self-consciousness as the actualization of that capacity in our experience of the world beyond ourselves’ (2009, p. 99).^23^ Like the previous *via negativa* argument, this view suggests that the UIA-Backer has got something right—he has recognised that our capacity for inner awareness is bound up with all of our experiences. His mistake is to misconstrue the potential presence of inner awareness as an occurrent presence.

Again, the problem with this argument is that UIA-Backers are not generally inclined to mistake dispositional properties of experience for the actualisation of that property. At any given time, Dan Zahavi has the potential to report on his concurrent experience. But he is not (I assume) inclined to say that this potential is in some way actualised for all experiences: he does not, for instance, suggest that all experiences are accompanied by a background inner monologue describing that very conscious state. So why would he be making an analogous mistake in the case of his potential to gain inner awareness of his concurrent experience? The proposed explanation is at best incomplete. Interestingly, Schear recognises that there’s some further explaining to do here, so cites the fridge light fallacy to explain why UIA-Backers are vulnerable to a category mistake regarding inner awareness that they wouldn’t otherwise be inclined to make (2009, p. 101). As we have already seen though, the appeal to the fridge light fallacy fails to yield any dialectical traction. Yet again, the UIA-Skeptic is being far too uncharitable in her portrayal of the UIA-Backer, and so fails to offer a serious explanation of his phenomenological intuitions.

## An alternative model of inner awareness

Faced with a phenomenological disagreement over the ubiquity of inner awareness, UIA-Backers and UIA-Skeptics have each put forward a range of arguments designed to resolve the dispute. Having surveyed these arguments, it is clear that none of them has the dialectical purchase needed to break the phenomenological stalemate. In this section I attempt to break the stalemate by introducing an alternative model of inner awareness. The first sub-section makes a proposal about the place of inner awareness in our phenomenology. The second sub-section addresses three potential objections to this proposal. The third sub-section suggests that this proposal offers a compromise position that accommodates some of the key claims made by each camp, and shows how it is equipped to offer a credible and charitable explanation of why the entrenched camps get their own phenomenology wrong.

### The Affordance Model

If we wish to give a rich and accurate description of our phenomenology, we need to have the notion of affordances in our conceptual toolbox. When we perceive the world we are not merely passive spectators but rather active participants. Our potential to engage with our environment figures in our perceptual experience. The ball is not just given to us as red and round, it is given to us *as kickable*. We don’t just experience the traffic light as green, we experience it *as inviting acceleration*. We don’t just perceive Justin Bieber’s face as babyish and self-satisfied, we perceive it *as slappable*. In all these cases, the object we perceive appears to *afford* a certain action—eating, kicking and slapping respectively. Our opportunity to perform these acts figures in our experience: there is a manifest phenomenological difference between just seeing the ball and seeing it *as kickable*.

Our phenomenology is coloured by apparent affordances.^24^ As with any apparent property, the appearance of an affordance is doubly dissociable from the actual presence of that affordance. You can be in a position to perform an act without this opportunity being apparent to you: you can be in a position to kick a ball without *experiencing* it as kickable. Conversely, in a non-veridical experience of an affordance, an opportunity to act seems to be available to you when it is not: a ball that *seems* kickable might actually be made of lead. As with perceptual appearances, experiencing an affordance should not be conflated with *judging* there to be an opportunity for action. Believing that you can kick an object is one thing, but *experiencing it as kickable* is quite another. Merely attributing the belief to a subject would fail to capture how *kickability* figures in her phenomenology. This comes out when we consider the possibility of *experiencing* the ball as kickable whilst simultaneously *judging* it not to be. Affordances also shouldn’t be conflated with *imaginings*. You don’t have a perceptual experience of the ball plus an imaginative experience of yourself kicking the ball. Rather, the kickability of the ball figures immanently in your experience of the ball itself.^25^

Our experience of affordances can vary depending on the skills we have acquired. For instance, we begin to experience the green traffic light as inviting acceleration only after acquiring the ability to drive.^26^ By contrast, affordances of grasping are often thought to figure in the perceptual experience of neonates. Our experience of affordances can also vary depending on the extent to which our environment *solicits* us to act (see Siegel 2014). Perhaps a collection of balls before you each seem kickable, but one particularly well-placed ball *calls out* to be kicked, colouring your experience in a way that the other balls do not.

Armed with the concept of affordances, we can offer a novel account of how inner awareness figures in our ordinary non-introspective experiences. Introspection is an action. All conscious states—or at least all ordinary conscious states of normal adult humans—are introspectable. This is a claim that both UIA-Backers and UIA-Skeptics seem to be able to get behind (see especially Thomasson 2005; Kriegel 2009a). My suggestion is that this ever-present potential for introspection actually figures in our experience. Your capacity to gain inner awareness of your concurrent conscious state colours *what it is like to be* in that state for you. Although our outer awareness of the world is not generally accompanied by an inner awareness of that very state, it is accompanied by an awareness of the *opportunity* for introspection. In other words, *an affordance of introspectability is a ubiquitous feature of our phenomenology*.

Thomasson comes close to this proposal when she suggests that ‘…it is a continuous part of our experience that we are immediately able to report on such experiences…’ (2006, p. 7). Unlike Thomasson though, my proposal concerns the potential for introspection rather than the potential to report on one’s experience. I am also not committed to Thomasson’s theory of how introspection and phenomenological reports work. In fact, this ‘Affordance Model’ can be combined with whatever is your preferred theory of introspection. So long as introspection is an act, we can experience an affordance to introspect, regardless of the details of what this act involves.^27^ The Affordance Model is also consistent with introspection being a bundle of inter-related cognitive capacities rather than a unitary skill. If this were the case, we should expect a collection of subtly different affordances corresponding to the different cognitive activities.

Presumably infants and non-human animals cannot introspect. Abnormal humans, such as blindsighters, might be attributed unintrospectable conscious states. In normal humans the capacity for introspection might go ‘offline’ during abnormal states of consciousness such as dreaming. In all of these cases, it is unlikely that the subject’s experience is characterised by an affordance of introspectability. The Affordance Model does not make a universal generalisation about conscious states that would include such atypical experiences. It does, however, seek to make a generalisation about all ordinary experiences of normal adult humans. The ordinary experiences of normal adult humans are introspectable, and the introspectability of those experiences is phenomenologically manifest to the subject.^28^

### Objections and replies

I will now consider three potential objections to the Affordance Model and argue that they can each be avoided given appropriate clarification of the proposal. The first objection is that affordances correspond to potential bodily actions. When Justin Bieber’s face affords slapping, for instance, the phenomenology of this experience is bound up with a specific possible movement of one’s arm. But since there is no bodily action involved in introspection, there can be no affordance of introspectability. Against this objection I would reply that there are many cases of affordances for *mental* as opposed to bodily actions: a loud noise can afford attention, a lecture can afford concentration, and a statement can afford contemplation. Affordances of introspection are members of a larger group of *cognitive* affordances. A critic might respond that these cases cannot properly be described as ‘affordances’. However, the notion of cognitive affordances is not without precedent in the literature (e.g. Scarantino 2003, p. 960). And even if the critic insists that affordances must involve bodily actions, at this point I think we would have a merely terminological dispute. If our capacities to introspect, attend, concentrate and contemplate can colour our phenomenology even when we are not deploying those capacities, then the phenomenological features in question are affordances in every way that matters to me. If there are technical reasons to regard them as features *analogous* to affordances rather than as affordances-proper, the real content of my proposal would be unaffected.

The second objection concerns the sophistication that an affordance of introspectability would need to have. To be aware of our capacity to introspect, we would need to be aware of ourselves as agents with the ability to reflect on our experience. But in ordinary experience we are not aware of ourselves, or at least not aware of ourselves in any sophisticated way. Therefore in ordinary experience we are not aware of our capacity to introspect. This objection rests on an excessively intellectualised understanding of affordances. The ball can seem kickable without you forming any sophisticated judgment about yourself as a potential kicker. In fact, it’s quite credible that it could seem kickable even to creatures with no concept of kicking i.e. no capacity to entertain propositions about kicking. The way that kickability contributes to our experience is relatively unsophisticated. By the same token, the way that introspectability contributes to our experience is also not that sophisticated. Perhaps experiencing affordances requires us to experience ourselves as agents—as entities that can deploy their abilities—but I don’t see any reason to regard this as too sophisticated for ordinary non-introspective consciousness.^29^

The third and final objection goes a little deeper but can still be overcome. If an act appears to be afforded to us, there must be something we are aware of which appears to afford that action. When kicking is afforded, for example, there must be a specific object that seems kickable to us. We never have a free-floating sense of *kickability* detached from any particular apparent object. What is it, then, that affords introspection? Here the Affordance Model seems to face a dilemma. One option is to say that it is *something in the world* that affords introspection. But the difficulty here is that external objects are not credibly introspectable. You cannot, for instance, introspect a red ball. On this horn of the dilemma, the Affordance Model is committed to an untenable view of introspection. The other option is to say that the thing that affords introspection is your conscious state. You cannot introspect a red ball, but you can introspect your *experience* of the red ball. But the difficulty here is that experiencing an affordance of introspectability would then presuppose an awareness of the state to be introspected. Consequently, if all our experiences afford introspection then we must be aware of all our experiences. On this horn of the dilemma, the Affordance Model thus collapses into UIA.

The dilemma trades on the following phenomenological principle: *if an action φ appears to be afforded then there must be some apparent object x that appears to afford having φ done to it.* I will seek to undermine this principle, thus allowing the Affordance Model to avoid the dilemma. The easiest way to find counter-examples to this principle is to consider acts that, unlike kicking or slapping, are not directed at an object. Dancing, for example, is not something we do *to* an object (Siegel 2014). Accordingly, when dancing appears to be afforded there is no object that appears to afford having dancing done to it. When you experience an affordance to dance in a nightclub, it is the *situation—*the music, the lights, the right degree of intoxication—that presents an opportunity for dancing. My suggestion is that introspection is like dancing in this respect. We can be aware of the opportunity to introspect in our current situation without having to be aware of any object *as a thing to be introspected*. Unlike with dancing, *every* ordinary situation presents an opportunity for introspection which is why the affordance of introspectability is a ubiquitous feature of our phenomenology.

Here the critic might respond that introspection is not relevantly analogous to dancing. Dancing is a counter-example to the original phenomenological principle because dancing is not an action directed toward an object. Introspection, however, is directed toward an object: specifically, one’s concurrent experience. As such, we should expect affordances of introspectability to have the same phenomenological structure as affordances of other object-directed actions. In other words, the critic can retreat to the following qualified version of the phenomenological principle: *for all and only object*-*directed actions*, if an action φ appears to be afforded then there must be some apparent object x that appears to afford having φ done to it. Since introspection is indeed an object-directed action, this qualified principle suffices to underwrite the dilemma for the Affordance Model.

My response to this objection is that even the qualified phenomenological principle doesn’t hold. Consider the act of searching for shelter. This is clearly an object-directed action: it is aimed toward the shelter. Presumably there are situations in which this action is afforded, but it is implausible that being aware of this affordance requires being aware of the object that is to be searched for. After all, a subject can search for shelter only if she is yet to find it, but can be aware of the shelter only once it has been found.^30^ I suggest that affordances of introspection are analogous to affordances of shelter-searching. In both cases, the subject is aware of the opportunity to perform an object-directed action without being aware of the object in question. And in both cases, the subject only *becomes* aware of the object in question if she successfully performs the afforded act. The lesson we learn from the shelter-searching case is that if an action *reveals* an object to the subject, then we should not expect experiences of an affordance to perform that act to be characterised by an awareness of the object to be revealed. Introspection is just such an action: performing it reveals our own experience to us. As such, we should not expect the experience of introspectability to be characterised by a prior awareness of the conscious state to be introspected.

### The dialectical significance of the Affordance Model

Ultimately the Affordance Model is answerable to your phenomenological reflection. If careful investigation suggests to you that this account accurately describes your experience, then you won’t need any theoretical arguments to convince you of its truth. If no amount of investigation makes this description plausible, then the theoretical arguments I offer may not persuade you. Nevertheless, it is worth considering how the Affordance Model can weigh in on the debate between UIA-Backers and UIA-Skeptics.

One thing to note is that the Affordance Model qualifies as a UIA-Skeptic position: it denies that all experiences of normal adult humans are characterised by an inner awareness of that very experience. That said, standard forms of UIA-Skepticism (i.e. all sceptical positions other than the Affordance Model) do not posit a ubiquitous affordance of introspectability. In fact, by making such a posit the Affordance Model reflects something of the spirit of the UIA-Backers. Overall, the Affordance Model is best regarded as a *compromise position* that finds a middle ground between the UIA-Backers and the standard UIA-Skeptics. It claims that both sides are partly right and partly wrong, and that the truth lies somewhere between them. I hope to show that by adopting this reconciliatory posture, the Affordance Model can gain far greater dialectical traction than either of the two entrenched camps.

What exactly does the Affordance Model say about the merits and failings of the two entrenched camps? It says that the UIA-Backer is *right* to deny that the phenomenology of non-introspective experiences is exhausted by our outer awareness, but *wrong* to say that those experiences are characterised by an occurrent inner awareness. Occurrent inner awareness is in fact limited to episodes of introspection. On the other hand, the standard UIA-Skeptic is *right* to deny that non-introspective experience is characterised by occurrent inner awareness, but *wrong* to say that the phenomenology of non-introspective experience is exhausted by outer awareness, with inner awareness making no contribution to our experience. Although inner awareness is not always present, its *potential* to be brought into presence is in fact a ubiquitous feature of experience.

Now, since the Affordance Model says that both of the entrenched camps are partly mistaken, it owes us an explanation of why these mistakes are made. My suggestion is that both camps misdescribe their phenomenology because they lack the concepts needed to provide a fully accurate description. When trying to describe our experience, we are somewhat restricted by the phenomenological concepts with which we are familiar. One concept that neither camp has at its disposal is that of *affordances of introspectability*. Without this concept, it is easy for one’s phenomenological judgments to be led astray. Once the UIA-Skeptic has correctly recognised that non-introspective experiences do not involve occurrent inner awareness it is natural, though mistaken, for them to infer that our capacity for inner awareness can have no influence on what it’s like to undergo such experiences. Similarly, once the UIA-Backer has correctly recognised that our capacity for inner awareness does influence what it’s like to undergo non-introspective experiences it is natural, and again mistaken, to infer that inner awareness is occurrently present in such experiences. Neither camp is in a position to frame the possibility that our capacity for inner awareness contributes to our phenomenology by presenting an *affordance* for us to deploy that capacity, rather than by actually being *deployed.* The phenomenological descriptions offered by both camps are imperfect *approximations* of the truth: they reflect people’s best effort to describe their experience with the limited concepts at their disposal. Of course, by introducing the Affordance Model I hope to correct this mistake.

Throughout the paper I have contended that the arguments presented by both UIA-Backers and standard UIA-Skeptics lack genuine dialectical force. In what ways does the Affordance Model improve on the entrenched positions in this regard? One of the main objections I’ve raised is that each camp fails to take the phenomenological intuitions of their opponents seriously enough. The Affordance Model has the advantage of taking the intuitions of both sides very seriously. Although it says that the UIA-Backers are wrong, it regards them as closer to the truth than the standard UIA-Skeptic regards them. Similarly, it says that the standard UIA-Skeptics are wrong, but regards them as closer to the truth than the UIA-Backer regards them. Like any compromise proposal, the Affordance Model manages to avoid an excessively uncharitable view of either camp in the debate.

A further objection I’ve raised is that neither of the entrenched camps has successfully explained why their opponents would get their own phenomenology wrong. Specifically, I argued in Sect. 3 that none of the *via negativa* arguments offered satisfy the following two rules: I) not being applicable against both camps with equal force and; II) not attributing the opponent a kind of mistake that she is not otherwise disposed to make. The Affordance Model holds that both camps partly misdescribe their phenomenology because they lack the concept of *affordances of introspectability*. This proposal satisfies the first rule because a critic cannot respond that it is actually proponents of the Affordance Model who have been misled by their more limited conceptual repertoire. The Affordance Model adds a new concept into the existing mix, so it cannot be the case that it is proponents of the entrenched views that actually have the richer conceptual repertoire. The Affordance Model also satisfies the second rule. When we lack the concepts needed to fully describe a phenomenon, we are generally vulnerable to the mistake of partly misdescribing that phenomenon. Specifically, we are all disposed to make the mistake of offering imperfect approximations of the truth using the concepts at our disposal. Consequently, the Affordance Model does not attribute either camp a kind of error that they are not otherwise disposed to make. In fact, it charitably suggests that the mistakes made by all parties are relatively innocent. Overall then, the Affordance Model is in a far better position to explain the mistakes of its opponents then either of the entrenched camps is.^31^

Does this explanation of where the entrenched camps have gone wrong commit me to the prediction that on reading this paper they will immediately see the error of their ways and become card-carrying supporters of the Affordance Model? I am not optimistic about the prospects of this ambitious prediction, but fortunately the Affordance Model is not committed to such a forecast. Sometimes people do not abandon their mistaken judgements even when the source of their mistake is correctly identified and a superior alternative position is presented to them. If proponents of the standard views do not abandon their positions when presented with the Affordance Model, this would be quite consistent with the model having diagnosed their mistakes accurately. Perhaps the Affordance Model could be asked to provide a *further* explanation here—an account not just of why people make their initial mistaken judgement but of why they retain it after it has been undermined. Such a further explanation should not be hard to come by. Perhaps proponents of the standard views are too entrenched in their mistaken ways of thinking to recognise the value of the alternative with which they are presented. Philosophy is replete with cases of this kind. Or perhaps they don’t yet have a sufficient understanding of the alternative view to see its truth. As we have already seen, it is difficult to capture what an affordance of introspectability would be like, so dissenters could easily be looking for *the wrong thing* in their experience.

 The question of whether inner awareness is ubiquitous has led to a recalcitrant phenomenological disagreement. Both sides of the dispute have tried to break this stalemate by deploying a number of arguments against their opponents, but we have seen that none of these arguments has any real dialectical purchase. Transcending these entrenched positions, the Affordance Model offers a fresh account of the relationship between our capacity for inner awareness and the phenomenology of non-introspective experience. At the very least this model offers a new avenue of enquiry. Beyond that though, I hope to have shown that it has significant dialectical advantages over its competitors. It offers a compromise position that respects the driving phenomenological insights of both camps, and provides a charitable explanation of where each side has been led astray.

## Footnotes

1 UIA-Skeptics can still hold that there is a constitutive connection between consciousness and awareness (Gertler 2012, p. 451). The claim they are resisting is that conscious states must be characterised by an awareness *of that very state*.

2 Consequently, inner awareness must not be confused with making judgements about one’s concurrent experience. In principle, you can have a judgement-free awareness of your experience just as you can have a judgement-free awareness of your environment.

3 Some UIA-Backers such as Zahavi (2004, 2006) claim that awareness of one’s experience does require a kind of awareness of oneself, though this awareness involves a primitive sense of ‘mineness’ rather than a rich conception of the self. This issue is helpfully discussed by Zahavi and Kriegel (forthcoming). For current purposes I can remain neutral on this claim.

4 I take it that the crucial distinction between introspective and non-introspective awareness can be made without committing to any particular theory of introspection. The important point is that there is a *phenomenological* difference between what it’s like to have the non-introspective awareness cited by UIA-Backers and what it’s like to reflect explicitly on your experience.

5 Zahavi holds that all the major figures in the phenomenological tradition—Brentano, Husserl, Heidegger, Sartre, Merleau-Ponty, Ricoeur—advocated some version of this view (2004, p. 82), though there is room for debate on Zahavi’s reading of these figures. Is Kriegel’s ‘peripheral inner awareness’ the same thing as the phenomenologists’ ‘pre-reflective self-consciousness’? There are some subtle differences between the two accounts (see especially Kriegel 2009a, p. 362; Zahavi 2004, pp. 79–80; Zahavi and Kriegel forthcoming). For instance, while the former is characterised as an intentional relation the latter is generally regarded as non-intentional. However, rather than getting side-tracked by this fiddly internal debate, I will treat Kriegel and the phenomenologists as allies who wish to defend at least some version of UIA.

6 Kriegel (2009a, pp. 126–127) argues that his proposal avoids any such regress but critics such as Levine (in conversation) argue otherwise. Faced with these worries a one-state theorist might hold that conscious states have an unstructured reflexivity. The worry here though is that the prospects of naturalising reflexive mental representations that lack internal structure are dim (Kriegel 2009a, p. 102).

7 One might instead make a *conceptual* case for UIA, arguing that inner awareness is built into the very concept of consciousness. I am concerned only with the phenomenological route to UIA, and doubt that a serious conceptual case can be made.

8 Note, the claim that we are easily able to make reliable reports about our experience is consistent with these reports sometimes involving errors. There is no presupposition of infallibility.

9 Effectively the same argument is made by Kriegel (2009a, p. 305).

10 Although the ‘nothing dramatic’ datum is clearly linked to the ‘no surprises’ datum, it remains important to distinguish them. There being ‘nothing dramatic’ about introspection pertains to how the act of introspection feels when we perform it. By contrast, there being ‘no surprises’ in introspection pertains to the epistemic relationship we appear to have to the introspected experience revealed by that act.

11 A possible alternative strategy for the UIA-Skeptic is to deny that UIA is even able to account for the data. If UIA doesn’t offer a genuine explanation of the data, then UIA-Skeptics are not under any obligation to provide a *better* explanation. Such a pessimistic view of UIA is hard to defend, and I cannot find any UIA-Skeptics who have adopted this view. Zahavi and Kriegel (forthcoming) attribute such a view to Schear (2009), but on my reading of Schear he makes no such claim.

12 The UIA-Backer might respond that the only way we could have knowledge of our concurrent experience is if that experience was characterised by an inner awareness (Caston 2006; Zahavi and Kriegel forthcoming). This is, however, a bold theoretical claim that is too contentious to be deployed with any real force against the UIA-Skeptic.

13 Should this knowledge be characterised in terms of an occurrent non-conscious mental state representing one’s concurrent experience, or in terms of a latent disposition to form such a representation when prompted? Schear (2009, p. 100) appeals to ‘…an ordinary colloquial sense of the verb ‘to know’…’, which I take to be neutral on the occurrent/dispositional question. Zahavi and Kriegel (forthcoming) argue that the dispositional account is explanatorily vacuous. If this objection sticks, the UIA-Skeptic can specifically commit to the occurrent-state version.

14 A subtly different UIA-Skeptic account of the ‘no surprises’ datum is offered by Gertler (2012, pp. 452–453). Though credible, it trades on contentious claims about qualitative awareness that would lead us too far astray.

15 This view of introspection is closely tied to the so-called transparency of experience. Many believe that when we introspect we do not become aware of some new set of qualities belonging to our experience. Rather, we become aware of the same old worldly qualities in some new way. The transparency of experience is sometimes thought to be at odds with UIA (e.g. Gennaro 2008), but Zahavi and Kriegel (forthcoming) go some way toward assuaging these worries.

16 Confusingly, Husserl and Thomasson use the concept of bracketing as part of a rejection of ‘introspectionist’ views of how we achieve knowledge of our experience. They take ‘introspection’ to be a quasi-perceptual inner observation, and rightly hold that bracketing is not introspective (see Thomasson 2005, p. 116). However, in this paper I am using the term ‘introspection’ more liberally to refer to the act that we perform—whatever it is—when we reflect on our experience. On this use of the term, it is quite coherent to say that the act of bracketing is one and the same as the act of introspection.

17 One interesting alternative to the bracketing account is Dretske’s (1994) ‘displaced perception’ model of introspection that shares the spirit of Husserl and Thomasson’s position but avoids the slightly contentious notion of bracketing. A further alternative would be to hold that introspection feels undramatic because it is the act of bringing into consciousness knowledge that was already present unconsciously, and not an act that yields any new object of knowledge. This would tally nicely with the responses offered to the first two pieces of data discussed.

18 Other *via positiva* arguments for UIA are available beyond the three influential arguments I have considered (see especially Kriegel 2009a). I suggest that these arguments fall short in the same way as those discussed: whatever data is cited in favour of UIA, an alternative skeptical explanation of the data is always available, so the dialectical stalemate persists.

19 I take it that this *via negativa* argument is implicit when UIA-Skeptics suggest that belief in UIA is driven by particular theoretical mistakes (e.g. Thomasson 2006, p. 7).

20 The same argument is made by Gennaro (2008, pp. 48–49).

21 Thomasson leans this way too (2006, p. 8) though her comments fit better with the fourth *via negativa* argument discussed.

22 This is compatible with the subject always having an occurrent *non*-conscious knowledge of her concurrent conscious state, though this is by no means a commitment of the proposal under consideration.

23 Mehta puts forward a subtly different proposal in the same vein when he suggests ‘…it is easy to become confused between being aware of the properties which put me in a position to become aware of my experience, and actually being aware of my experience’ (2013, p. 369).

24 The term ‘affordance’ was introduced by Gibson (1979). On Gibson’s view, to perceive an affordance is to stand in a direct non-representational ‘pick up’ relation to it. This controversial view of perception is difficult to unpack and hard to defend. Fortunately though, Gibson’s concept of affordances is easily decoupled from his idiosyncratic view of perception (Scarantino 2003, p. 954; Prosser 2011, p. 481). Following Prosser (2011), Siegel (2014) and others I will assume that perceptual experiences are representational, and that an affordance contributes to our phenomenology just in case our perceptual state represents the presence of that affordance. This is not the only way in which my understanding of affordances will diverge from Gibson’s. I would note, though, that Gibson himself regarded the concept of affordances as open to improvements (see Sheehy 2004 quoted in Jenkins 2008, p. 43) and that current commentators still regard the meaning of the term as somewhat up for grabs (Scarantino 2003, p. 949; Jenkins 2008, p. 43). As such, I will not worry about the extent to which my understanding of affordances qualifies as ‘Gibsonian’.

25 Some might be uncomfortable with the suggestion that affordances are perceptible properties. Affordances should be regarded as dispositional properties (Scarantino 2003), and it could be claimed that dispositional properties are not the kind of property we can perceive. One option here is to deny that affordances are dispositional (Caiani 2014), but my preferred route is to accept that they are dispositional but deny that dispositional properties are imperceptible. Perceptual experience appears to present us with dispositional properties (including, but not limited to, affordances) so the burden of proof is on the opposition to demonstrate that dispositional properties are, despite appearances, imperceptible.

26 For discussion of such ‘emergent’ affordances see Dotov et al. (2012). Another factor that might influence our experience is the extent to which we have *mastered* a skill: the affordances presented to a new driver will be very different to those presented to a professional racer.

27 If there is *no such thing* as introspection then the Affordance Model would be in trouble. I take it though that when theorists deny the existence of introspection, what they are really rejecting are certain naïve models of how introspection works (see footnote 16 above).

28 When we introspect, our introspective experience can itself be introspected. As such, I am open to the possibility that even introspective experiences are characterised by an affordance of introspectability, though little seems to ride on this.

29 A critic might insist that experiences of affordances must be underwritten by exclusively low-level psychological processes. Since awareness of our ability to introspect would presumably require high-level cognitive processes, there can be no affordances of introspectability. In response I would note that there is no reason to think that the psychological processes that underwrite awareness of affordances are uniform (see Scarantino 2003, pp. 960–961). The perception of classic affordances like kickability might be accounted for entirely in terms of low-level processes, while other affordances like introspectability involve high-level processing.

30 I am assuming that the thing we are *searching for* is the object of our act of searching, but others might instead think that the thing *being searched* is the object of our act. If this is so, it becomes more plausible that experiencing an affordance of searching for shelter requires us to be aware of the object of our action (viz. the thing to be searched). For instance, if I am searching for shelter in a building then I will be aware of *the building* that I am searching even if I am not yet aware of the suitable sheltering place within it. It is not quite clear how to describe such cases, but I would argue that searching can be afforded even if we are unaware of any particular thing *to be searched*. When caught in stormy weather, shelter-searching can be afforded to me even if I am wholly unaware of any particular object to search (perhaps because it’s very dark). This would constitute a counter-example to the revised phenomenological principle.

31 An interesting further possibility is that the Affordance Model can be used to *supplement* some of the more promising *via negativa* arguments already on offer. The fourth *via negativa* argument offered by the UIA-Skeptics in Sect. 3 suggested that UIA-Backers confuse the ubiquitous *potential* for inner awareness with the ubiquitous *actualisation* of that potential. My objection to this argument is that the skeptic fails to explain why the UIA-Backer would confuse a dispositional property for its actualisation when he is not otherwise inclined to make this mistake. The Affordance Model may be able to answer that question: apparent affordances are occurrent categorical features of your phenomenology. Confusingly though, these categorical occurrent properties represent the presence of some dispositional property. For instance, it is a categorical feature of your experience that it presents the ball to you as kickable, but what this represents is the *potential* for kicking and not the actualisation of that potential. So perhaps the UIA-Backer has noticed the following phenomenon—*the categorical presence of a property representing their potential to gain inner awareness*—but misdescribed it as the categorical presence of inner awareness. Unlike other dispositional properties of experience, introspectability has an occurrent phenomenological proxy in the form of an affordance, and it is this that makes the UIA-Backer vulnerable to a category mistake that he wouldn’t otherwise make.

## References

[CR1] Armstrong DM (1968). A materialist theory of mind.

[CR2] Brentano, F. (1874/1924). *Psychologie vom empirischen* (Standpunkt I. Hamburg: Felix Meiner; Trans; A. C. Rancurello, D. B. Terrell & L.L. McAlister). London: Routledge & Kegan Paul, 1973.

[CR3] Caiani SZ (2014). Extending the notion of affordance. Phenomenology and the Cognitive Sciences.

[CR4] Caston V (2006). Comment on A. Thomasson, “Self-awareness and self-knowledge”. Psyche.

[CR5] Dotov DG, Nie L, de Wit MM (2012). Understanding affordances. Avant.

[CR6] Dretske F (1994). Introspection. Proceedings of the Aristotelian Society.

[CR7] Gennaro RJ (2008). Representationalism, peripheral awareness, and the transparency of experience. Philosophical Studies.

[CR8] Gertler B (2012). Conscious states as objects of awareness: on Uriah Kriegel, Subjective consciousness: a self-representational theory. Philosophical Studies.

[CR9] Gibson JJ (1979). The ecological approach to perception.

[CR10] Jenkins H (2008). Gibson’s “Affordances”: Evolution of a pivotal concept. Journal of Scientific Psychology.

[CR11] Kriegel U (2009). Subjective consciousness: A self-representational theory.

[CR12] Kriegel U (2009). Self-representationalism and phenomenology. Philosophical Studies.

[CR14] Mehta N (2013). Is there a phenomenological argument for higher-order representationalism?. Philosophical Studies.

[CR15] Prosser S (2011). Affordances and phenomenal character in spatial perception. Philosophical Review.

[CR16] Rosenthal DM (1986). Two concepts of consciousness. Philosophical Studies.

[CR17] Scarantino A (2003). Affordances explained. Philosophy of Science.

[CR18] Schear JK (2009). Experience and self-consciousness. Philosophical Studies.

[CR19] Siegel S, Brogaard B (2014). Affordances and the contents of perception. Does perception have content?.

[CR20] Thomasson AL, Smith DW, Thomasson AL (2005). First-person knowledge in phenomenology. Phenomenology and philosophy of mind.

[CR21] Thomasson AL (2006). Self-awareness and self-knowledge. Psyche.

[CR22] Zahavi D (2004). Back to Brentano. Journal of Consciousness Studies.

[CR23] Zahavi D (2006). Two takes on a one-level account of consciousness. Psyche.

[CR24] Zahavi, D., & Kriegel, U. (forthcoming). For-me-ness: What it is and what it is not. In D. Dahlstrom, A. Elpidorou, & W. Hopp, (Eds.) *Philosophy of Mind and Phenomenology: Conceptual and Empirical Approaches.* London: Routledge.

